# Supplementary material for: Anti-biofilm efficacy of a medieval treatment for bacterial infection requires the combination of multiple ingredients
Source: Sci Rep. 2020 Jul 28;10:12687. doi: 10.1038/s41598-020-69273-8 (PMC7387442; doi:10.1038/s41598-020-69273-8)
Supplement: Supplementary file 1 — Supplementary file1 (DOCX 7369 kb) [file 41598_2020_69273_MOESM1_ESM.docx]

**Anti-biofilm efficacy of a medieval treatment for bacterial infection requires the combination of multiple ingredients.**

Jessica Furner-Pardoe^1,2,+,^*, Blessing O Anonye^1,+^, Ricky Cain^1,#^, John Moat^3^, Catherine A. Ortori^4^, Christina Lee^5^, David A. Barrett ^4^, Christophe Corre^1,6^ and Freya Harrison^1,^*

^1^ School of Life Sciences, Gibbet Hill Campus, University of Warwick, Coventry, CV4 7AL

^2^ Warwick Medical School, Gibbet Hill Campus, University of Warwick, Coventry, CV4 7AL

^3^ Warwick Antimicrobial Screening Facility, School of Life Sciences, Gibbet Hill Campus, University of Warwick, Coventry, CV4 7AL

^4^ Centre for Analytical Bioscience, Advanced Materials and Healthcare Technologies Division, School of Pharmacy, University of Nottingham, Nottingham NG7 2RD, UK

^5^ School of English, University of Nottingham, Nottingham NG7 2RD, UK

^6^ Department of Chemistry, University of Warwick, University of Warwick, Coventry, CV4 7AL

^#^Present address: Evotec (U.K.) Ltd., 114 Innovation Drive, Milton Park, Abingdon, Oxfordshire OX14 4RZ, United Kingdom

^+^ These authors contributed equally

* Corresponding authors:

JFP (j.furner-pardoe.1@warwick.ac.uk) and FH (f.harrison@warwick.ac.uk)

**Supplementary Figures**


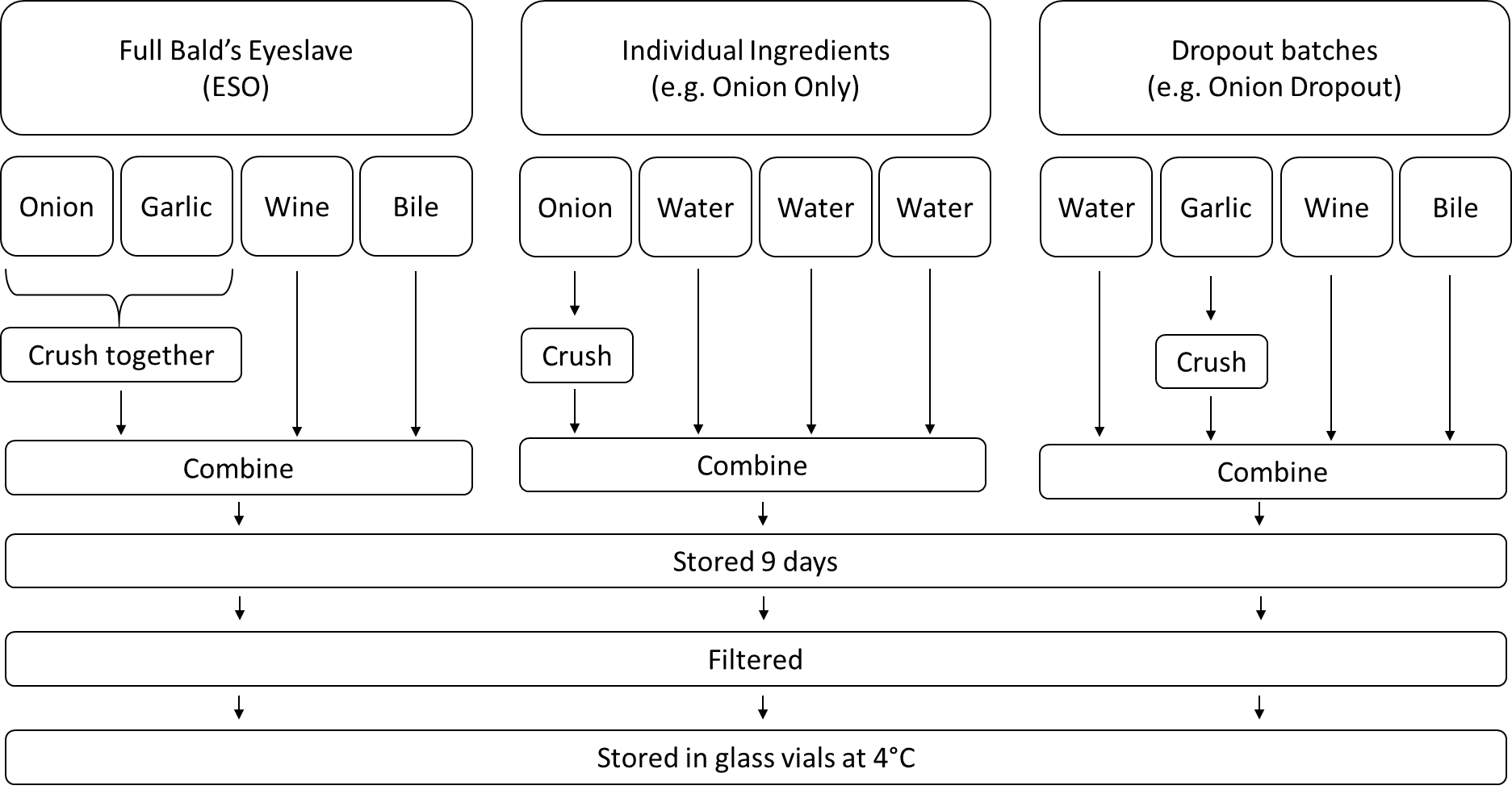


**Figure S1.** Schematic of the process to generate Bald’s eyesalve and various batch variations.

**
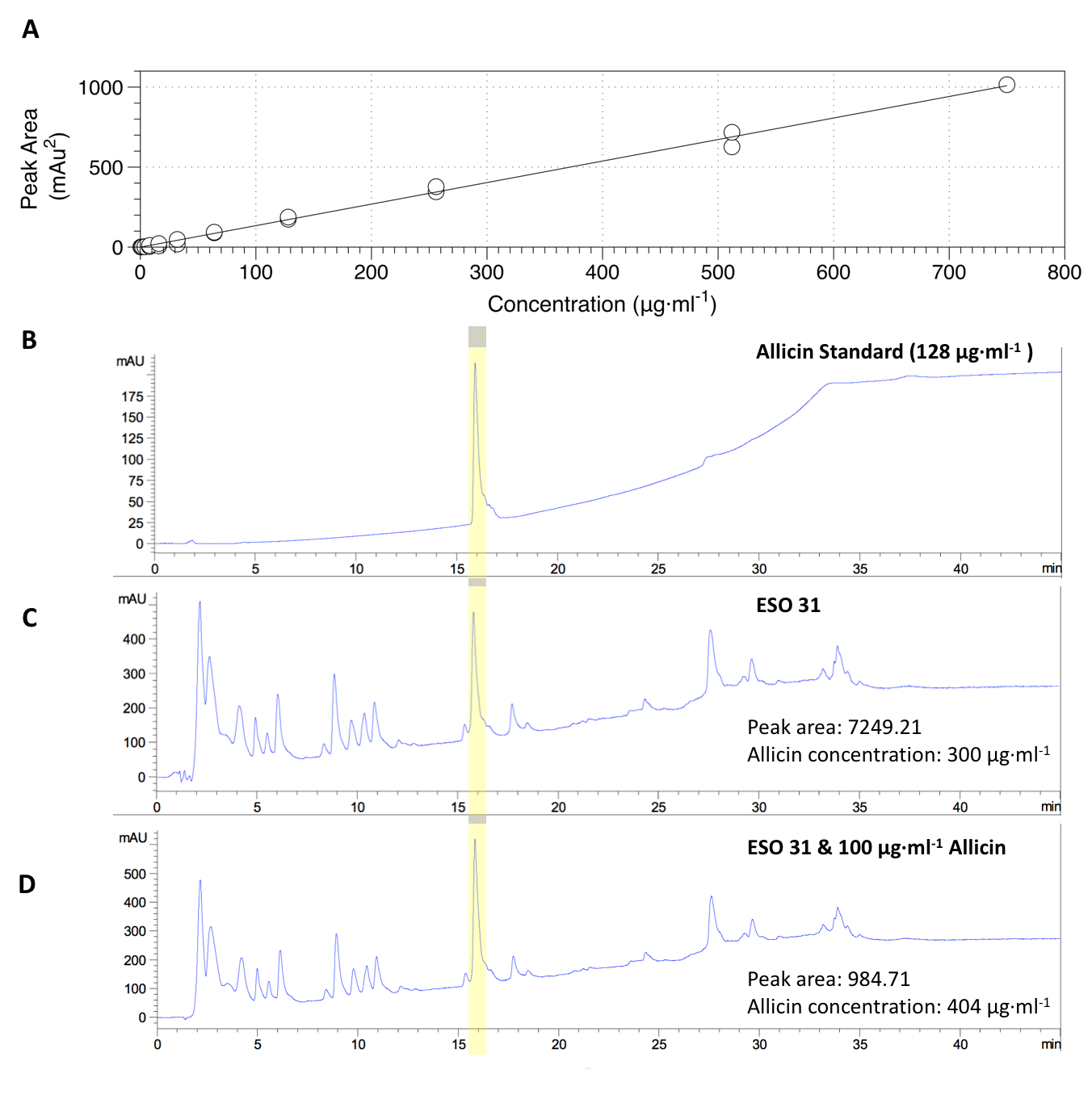
Figure S2. Confirmation of allicin in Bald’s eyesalve.** All chromatograms were run against a 0-95% methanol gradient, with a flow rate of 1 ml·min^-1^ on HPLC Aligent 1200 series, at 210 nm UV wavelength. A) Allicin calibration curve. External allicin standards, serially diluted in water were run and peak area was measured. A linear relationship was found between HPLC peak area and allicin concentrations between 8 - 750 μg·ml^-1^ with a correlation coefficient R^2^ value > 0.99. The mid-range of the curve (8-512 μg·ml^-1^) has 2 repeats. B) Exemplary allicin external standard (128 μg·ml^-1^) chromatogram, suspected allicin peak has a retention time of approx. 15 minutes. C-D) Bald’s eyesalve fresh batch, ESO 31 without (C) and with (D) an additional 100 μg·ml^-1^ of allicin standard; the differences in peak area are indicated. The highlighted region is the allicin peak.


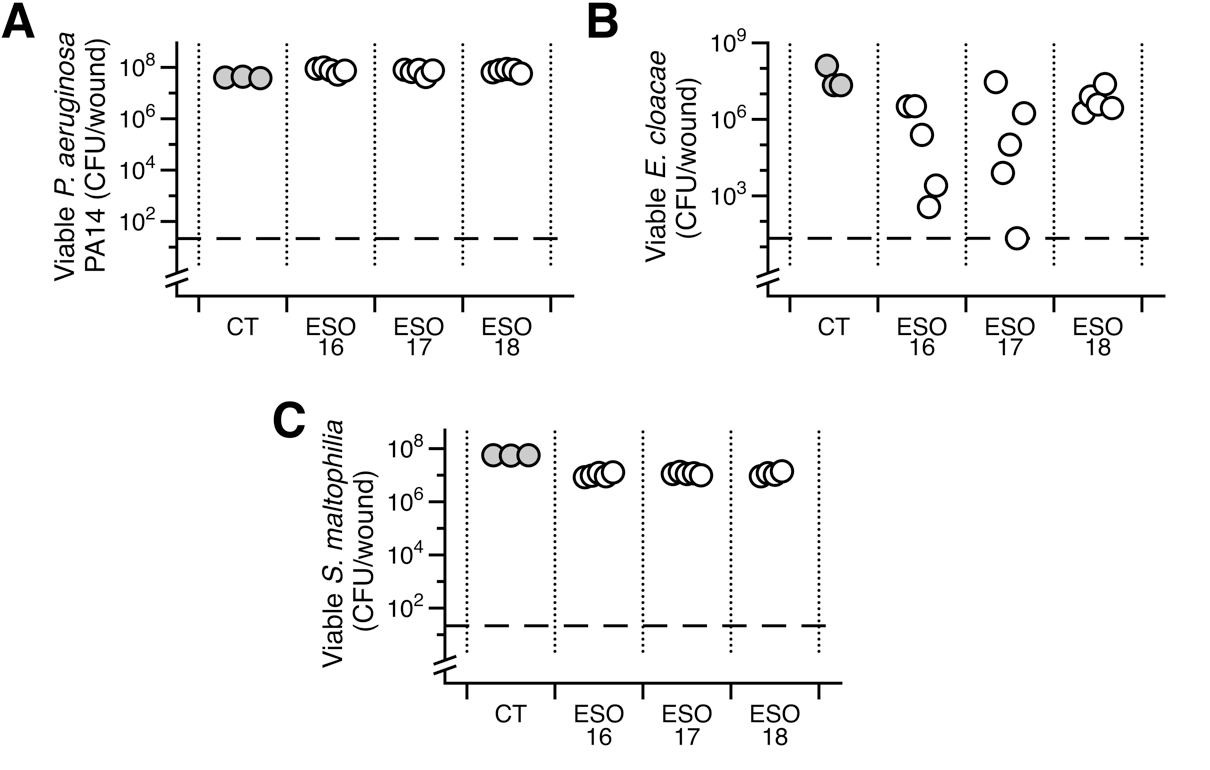


**Figure S3. Bald’s eyesalve (ESO) anti-biofilm activity.** Mature biofilms of various isolates were grown in a model of a soft tissue wound, then treated with either 0.5 vols sterile water (control, CT) or 0.5 vols of ESO for 24h before recovering bacteria for CFU counts (n = 3-5 replicates per treatment). The dashed line represents the limit of detection by plating. Data could not be transformed to fit assumptions of parametric tests, therefore, Kruskall-Wallis tests were used to determined that the CFU recovered from ESO-treated wounds was not significantly different from control wounds for *P. aeruginosa* PA14 (*X*^2^ = 6.88, df = 3, *p* value = 0.076), *S. maltophilia* (*X*^2^ = 7.63, df = 3, *p* value = 0.54) or *E. cloacae* (*X*^2^ = 7.74, df = 3, *p* value = 0.051). Raw data and R scripts are supplied in the Data Supplement.
